# Development of a hybrid mixed-mode solar dryer for product drying

**DOI:** 10.1016/j.heliyon.2023.e14144

**Published:** 2023-02-28

**Authors:** Arslan Afzal, Tahir Iqbal, Kamran Ikram, Muhammad Naveed Anjum, Muhammad Umair, Muhammad Azam, Sajeela Akram, Fiaz Hussain, Muhammad Ameen ul Zaman, Abid Ali, Faizan Majeed

**Affiliations:** a Faculty of Agricultural Engineering & Technology, PMAS Arid Agriculture University, Rawalpindi, Pakistan; b Department of Agricultural Engineering, KFUEIT, Rahim Yar Khan, 64200, Pakistan; c Department of Human Nutrition and Dietetics, University of Chakwal, 48800, Pakistan; d Department of Agricultural Engineering, Bahauddin Zakariya University, Bosan Road, Multan, 60800, Pakistan; e Department of Agricultural and Biosystems Engineering, University of Kassel, 37213, Witzenhausen, Germany

**Keywords:** Hybrid mixed-mode, Solar dryer, Quality analysis, Economic analysis, Peach (Freestone), Apple (Golden), Chili (Anaheim)

## Abstract

Sun drying in the open air is quite popular worldwide. However, the use of solar dryers to preserve various perishable agricultural products is a relatively new area of study, and the long-term effects of this method are not yet fully understood. The slow drying process in direct sunlight can contaminate the dried materials by soil and insects. To overcome these challenges, we devised a sun drying system that included a heating part, a drying area, a portable stand, fans, and a 50-W photovoltaic panel. An alternate energy source was used to power the drying process during cloudy days and at night. Fresh Freestone peach, Golden apple, and Anaheim chilies weighing 10 kg each with the initial moisture content of 89%, 87%, and 75% on a wet basis (w.b), respectively, were used in the experiments. The final moisture content of the samples was reduced by an average of 16%, 15%, and 11% for Freestone peaches, Golden apples, and Anaheim chilies, respectively. The quality analysis was carried out to determine sample composition, total bacteria, and color of dried products. The results indicated that the dried products met the recommended quality standards for food products in terms of composition, total bacteria, and color. This research supports the use of a hybrid mixed-mode solar dryer for drying a wide range of perishable agricultural products.

## 1 Introduction

Agriculture-based countries produce a wide variety of fruit and vegetables to feed the global population. The production of fruit and vegetables plays a vital role in food security, as these foods provide essential nutrients and contribute to a healthy diet. Agricultural countries play a crucial role in producing these foods, as they have the necessary land, resources, and expertise to grow and cultivate various crops. However, poor post-harvest handling and a lack of cold chain infrastructure can lead to a loss of 20–30% of agricultural productivity [[1], [2], [3]]. Common methods of food preserving include drying, canning, freezing, and pickling. Drying is the most common and economical method of preserving food without losing its nutritional value [4]. The shelf life of fruits and vegetables can be considerably improved by reducing their moisture content and water activity [5]. Drying is done to achieve a level of water content that inhibits microbial development and enzymatic and chemical processes [6]. Conventional drying methods, such as sun drying, have the disadvantages of nutrient loss and contamination [7]. In addition, the quality of dried food degrades over time when exposed to prolonged sun exposure [8]. Mechanical dryers are used for drying in a safe and controlled environment. However, mechanical drying is unfeasible in remote areas because of its high energy requirements [9].

The development and evaluation of solar-assisted dryers have been the subject of various studies. For instance, Singh and Gaur (2021) evaluated greenhouse solar dryer’s environmental effects and economic returns [10]. Selimefendigil et al. (2021) assessed the thermal performance of a solar-electric wood dryer. They also added a thermal air collector and heat recovery system to improve system efficiency [11]. Shreelavaniya et al. (2021) dried cardamom in a small solar-biomass hybrid dryer and discussed the drying kinetics, modeling, and quality of dried product [12]. Roratto et al. (2021) developed a hybrid solar vacuum dryer for different fruits and vegetables [6]. Nasri (2020) evaluated the thermal performance of a solar dryer for banana and peach fruits in Gafsa [13]. Borana (2020) conducted a study on a direct solar dryer. In two days, they dried a 20 kg batch of fresh tomatoes, potatoes, and chilies with a solar collector area of 1.03 m^2^ [14]. Sandali et al. (2018) developed a solar dryer that was coupled with a photovoltaic thermal generation system to dry tomato slices [15]. Mugi et al. (2022) increased the solar drying performance in the range of 9.5–47% by using materials like limestone, clay soil, sand, gravel, rocks, pebbles, and reinforced concrete [16]. Nukulwar et al. (2022) reduced the dying time by 9–16 h compared with the open sun dryer using a thermal energy storage system [17]. According to Atalay et al. (2022), a solar and wind-powered dryer can achieve an exergy efficiency of 68–89% [18]. A low-priced force convection solar dryer was developed by Lakshmi et al. (2019). The payback period for a solar dryer with an initial investment of $670 was calculated to be 0.65 years [19]. Philip et al. (2022) conducted an investigation of the technological and economic aspects of a greenhouse solar dryer. The payback period for the dryer was between 1.5 and 2.1 years, and the total cost was $2361 [20]. Tangne and Azese (2021) had developed a new firewood solar drier. According to their calculations, the dryer had an initial investment of $4600 and a payback period of 1.89 years [21].

Small farmers in developing countries often cannot afford the latest solar dryers due to their high costs [19,20]. While the efficiency of solar dryers has been extensively studied, there has been relatively little focus on reducing their cost. This study aimed to address this gap by developing a low-cost solar dryer with a uniform drying rate. To evaluate the effectiveness of the developed dryer, we tested it on three different agricultural products commonly produced in the rural areas of developing countries.

## 2 Materials and methods

### 2.1 Development of hybrid mixed-mode solar dryer

**Fig. 1:**
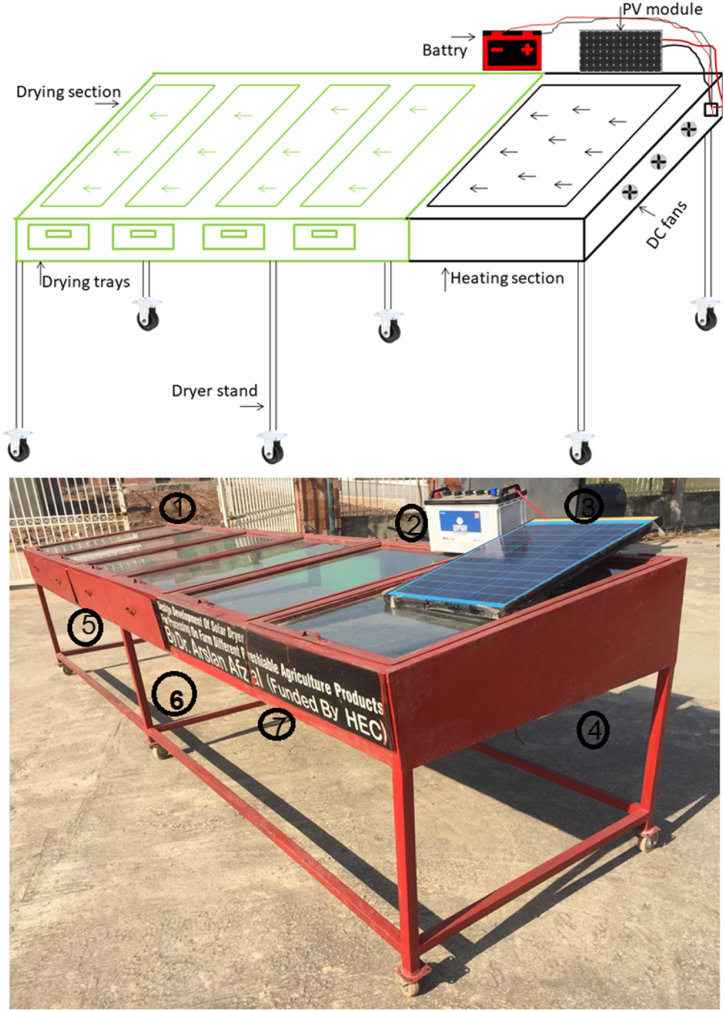
Fabricated hybrid mixed-mode solar dryer. 1- Drying section 2- Battery 3- PV module 4- DC fans 5- drying trays 6- Dryer stand 7- Heating Section.

This study was conducted at Pir Mehr Ali Shah, Arid Agriculture University, Rawalpindi, Pakistan. A modified solar dryer was designed and fabricated with mild steel sheets. A solar heater and three air-regulating fans were incorporated before drying trays; thus, the dryer was called a *hybrid mix-mode solar dryer* (HMSD). To reduce the dryer cost, locally available transparent glass was used to transmit and entrap solar energy in the heating section. The heating section was colored with H-28 metal black color. Three fans (with a 1000–1200 RPM speed each) were placed before the heating portion to ensure consistent airflow. Fans were powered from an attached solar photovoltaic plate; alternatively, electric power was also provided to continue the operation on cloudy days and at night. The HMSD (Fig. 1) contained a heating section, a mixed-mode drying section, a portable stand, three fans, a 50-W PV panel, and a battery.

### 2.2 Particulates of solar dryer

The newly designed HMSD was equipped with a heating portion that was 1.5 m in length. The mixed-mode operation was used to dry agricultural products due to the direct sunlight and hot air from the heating section. The drying section was 3 m long and contained four stainless trays (to reduce the moisture) with perforation for leaching water and to pass hot air to eliminate moisture from the bottom. Tray sieves were made of food grade nylon. Considering the geographical location, the dryer’s collection section (heating and drying) was tilted at 33° to get maximum solar intensity. Glass cover with a three mm thickness, having 95–98% transmittance, was used in the collection section. The dryer had a total width of one m and a cross-section area of 0.112 m^2^, with 36% assigned for heating and 64% assigned for drying. A 50-W PV panel was used to power the three DC fans at the bottom of the heating section during steady sunlight hours. The sunlight intensity (W/m^2^), humidity (%), drying temperature (°C), and air speed (m/s) were measured in the dryer using pyranometer, humidity and temperature sensors, and anemometers, respectively. Details of the instruments/material used in mix-mode solar dryer are shown in Table 1.

**Table 1:** Details of instrument/materials used in mix-mode solar dryer.

Sr. no.	Instrument/Material	Brand	Range
1	Pyranometer	LP02-05	±0.18 × 10^−6^ W/m^2^
2	Anemometer	UT363S	0.4–30 m/s
3	Temperature and humidity sensor	Mi BT	0–99 °C & 0–99%
4	PV panel	Longi	50 W
5	Glass	Local market	95–98% Transmittance

**Table 2:** Different parameters of experimental data.

**Time**	**Ambient Temperature (°C)**	**Collector exit Temperature (°C)**	**Change in Temperature (**ΔT)	**Relative Humidity R.H (%)**	**Collector exit Relative Humidity R.H (%)**	**Fan Speed (m/s)**	**Solar Radiations**$I_{c}$**(W/m**^**2**^**)**	**Thermal Efficiency (%)**
11:00	33	46	13	50	37	0.30	840	24.8
12:00	33	48	15	48	36	0.46	880	44.8
13:00	34	49	15	45	34	0.40	844	39.8
14:00	35	45	10	46	29	0.60	801	49.6

**Fig. 2:**
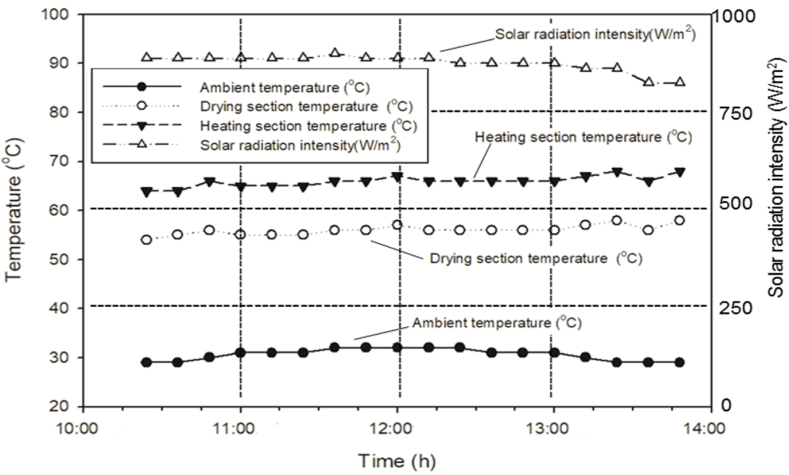
Performance of solar dryer on June 06, 2020, 30 readings averaged (during peak hours).

The solar intensity (W/m^2^), heating section temperature (°C), drying section temperature (°C), and ambient temperature (°C) as a function of time and temperature inside the solar dryer could be high, as shown in Fig. 2.

### 2.3 Provision of auxiliary energy source

**Fig. 3:**
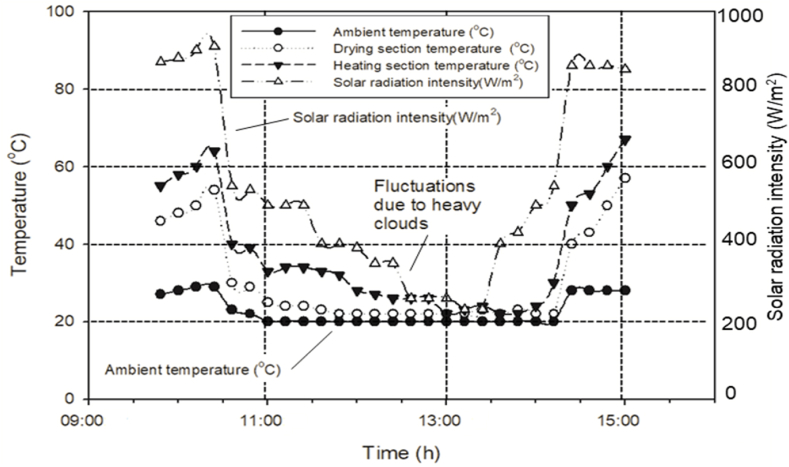
Solar radiation intensity, dryer inner, and ambient temperature (on July 09, 2020, 30 readings averaged).

The developed solar dryer was routinely operated in steady sunlight. However, several experiments require auxiliary energy sources because of poor solar intensity during cloudy days. Fig. 3 shows the effect of clouds on solar intensity, dryer internal temperature, and ambient temperature during day time in the summer season. A battery backup was attached to the dryer to power the DC fans during cloudy days or at night.

### 2.4 Preparation of sample

**Fig. 4:**
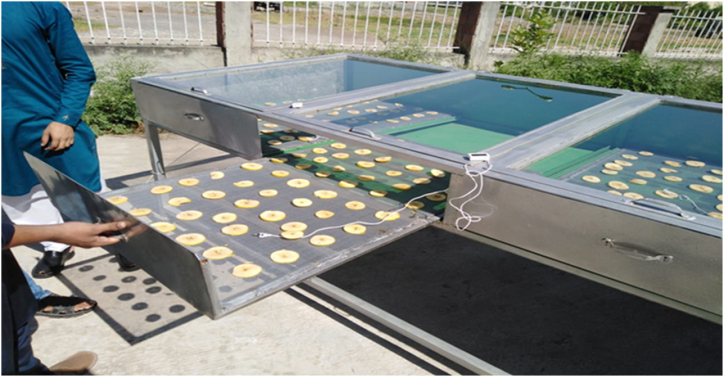
Loading apple slices in dryer trays.

Peach (Freestone), apple (Golden), and chili (Anaheim) were prepared for the drying experiment. Fresh products were purchased from the local market of Rawalpindi City. Ten kg of each fruit were purchased and transported to the Pir Mehr Ali Shah, Arid Agriculture University Rawalpindi. The selected fruit were transported with care to prevent any damage. After transportation, fruits were washed with tap water to remove any mud or dust particles. After that, fruit were delivered to the cooling section, and experiments were started. To ensure the accuracy of the results, ripped and uniform size fruit were selected for this study. Using a kitchen knife, three mm thick slices of apple were made as shown in Fig. 4. Peach was cut in to two halves to remove the nut. Chili was placed in the dryer without cutting. These agricultural products were treated with blanching method to stop the growth of bacteria and fungi attack. Balancing is the process in which food material is dipped into boiled water, removed after a 2–5 min period, and plunged into ice water.

Three replicates of the selected agricultural products were prepared, and each replicate was placed on dryer plates. Each plate contained 10–15 slices of the selected fruit, while chili pieces were placed approximately 25–50 mm apart to ensure the uniform application of air. About 10 kg of apple, peach, and chili were placed in the dryer. The heated air and the direct sunlight removed the moisture from the products. It took 12–18 h to complete the one trial. When solar intensity was low, the battery was used as a backup energy source to ensure a steady drying process.

### 2.5 Measurement of drying performance parameters

#### 2.5.1 Heat energy

The heat energy required to remove water from the agricultural product was calculated using Eq. (1) [21].

(1) $$H_{t}=W_{w}C_{p}\Delta T$$

where $H_{t}$ is the total heat energy required to remove water from the agricultural products in kJ, $W_{w}$ is the weight of water in the agricultural products, $C_{p}$ is the specific heat of air at constant pressure taken as 1.005 kJ/kgK^−1^, ΔT is the change in temperature taken as K.

Secondly, the moisture present in the agricultural products was evaporated. The heat energy required to evaporate the moisture from the agricultural products was calculated using Eq. (2).

(2) $$H_{e}=M_{w}\left(h_{g}{-h}_{f}\right)$$

where $H_{e}$ is the total heat energy required to evaporate moisture from the agricultural product in kJ, $M_{w}$ is the moisture content in the agricultural products, $h_{g}$ is the enthalpy of water as a vapor taken as 2583 kJ/kg, $h_{f}$ is the enthalpy of water as a liquid taken as 188 kJ/kg.

The heat absorbed by solar radiation in the collector was calculated using Eq. (3) [22].

(3) $$R_{i}=\infty A_{c}I_{c}$$

where $R_{i}$ the radiation heat absorbed by collector, ∞ is the absorptivity of the material, $A_{c}$ is the total cross-sectional area of the solar dryer in m^2^, and $I_{c}$ is the total solar intensity (W/m^2^) measured by the Pyranometer.

The heat loss by convection process from the collector was calculated using Eq. (4).

(4) $$C_{c}=\frac{\Delta T}{R_{o}}$$

where $C_{c}$ the heat loss by convection in the collector, $R_{o}$ is the thermal resistance across the thickness of sample (m). $R_{o}$ was calculated using Eq. (5).

(5) $$R_{O}=\frac{\Delta X}{A_{c}K}$$

where ΔX is the thicknesses of the sample used in the solar dryer, and K is the thermal conductivity of the sample (W/Km).

#### 2.5.2 Thermal efficiency

The total thermal efficiency of the solar dryer was calculated using Eq. (6) [23].

(6) $$\eta_{t}=\frac{{v\rho \Delta TC}_{p}}{A_{c}I_{c}}\times 100$$

where $\eta_{t}$ is the thermal efficiency of solar dryer in %, v is volumetric flow rate of air in m^3^/s, ρ is the air density in kg/m^3^. Thermal efficiency is the thermal performance of a solar dryer. The incoming solar radiations are not completely converted into heat; 2–5% of the radiations are reflected in the sky, while some are absorbed by glazing. Heat loss also occurs due to convection (absorbance) and radiations processes.

##### 2.5.2.1 Drying efficiency

The total drying efficiency of the solar dryer was calculated using Eq. (7) [24].

(7) $$\eta_{c}=\frac{Q_{E}}{Q_{i}}$$

where $Q_{E}$ is the total thermal energy gained or output in kWh of the solar dryer and $Q_{i}$ is the total incident thermal energy (or input thermal energy) of the solar dryer. The total thermal energy gained can be calculated by Eq. (8) [25].

(8) $$Q_{E}=mC_{p}\Delta T$$

where m is the total mass flow rate of air in kg/s. Total incident thermal energy can be calculated by Eq. (9) [26]

(9) $$Q_{i}=\frac{A_{c}I_{c}}{1000}$$

#### 2.5.3 Determination of drying rate

The drying rate is the rate at which moisture is removed from a given fruit sample, can be calculated by Eq. (10). The drying rate for the designed experiment was recorded by calculating the change in moisture content divided by the time taken to remove this moisture [27].

(10) $$Drying\;Rate\;\left(D.R\right)\;\left(\frac{g}{\min}\right)=\frac{Change\;in\;Sample\;Mass}{Time\;Taken}=\frac{M_{1}-M_{2}}{t_{1}-t_{2}}$$

where M_1_ and M_2_ are the initial sample moisture content, and final moisture content, respectively, (t_1_ - t_2_) is the time interval to remove moisture content.

#### 2.5.4 Determination of moisture content (M.C)

During drying, fruit slices were taken from the dryer to check the moisture content using a digital balance. Moisture contents of fruit slices were calculated on the wet basis by weight loss values. The moisture content of the samples was recorded after 15 min and noted as the average sample weight per unit weight of the fresh sample. Using Equation (11), moisture content was measured as the difference between dried and fresh samples. Moisture content on the wet basis was calculated using the protocols described by Chen et al. (2021) [28].

(11) $$M_{m}\left(w.b\right)=\frac{W_{O}-W_{d}}{W_{O}}$$

where $M_{m}\left(w.b\right)$ is the moisture content of sample on the wet basis, w_o_ and w_d_ are sample weights before and after drying, respectively.

#### 2.5.5 Quality analysis after drying of fruits

The dried agricultural products were analyzed using the following three tests.

I. Proximate test
II. Total plate count test (TPC)
III. Color test

Proximate analysis is a great approach for describing and assessing dried perishable agricultural items, according to Magalhaes et al., 2022. This method calculates the moisture, ash, crude protein, fat, and carbohydrate percentages in a dried sample. The proximate analysis was carried out according to the normal association of official chemists (AOAC) procedures [29].

The total plate count (TPC) test was used to calculate the total number of bacteria present in the sample, and this was done by following a previous study (Mailoa, Tapotubun, and Matrutty 2017). A 30–300 colony petri plate was used and sterilized in an oven at 180 °C for 2 h. To prevent the Petri dish from freezing after sterilization, the temperature was kept at 45–55 °C in a water bath. One litter aquadest and 8.5 g NaCl were used to make a dilute solution that was sterilized in an autoclave at 121 °C for 15 min. The dried sample was crushed and diluted in a dilute solution containing 10 g. The entire solution was poured into the Petri dish and incubated for 48 h at 37 °C. Bacteria multiply quickly for 48 h, after which the Petri dish was removed, and the bacterial colonies were counted [30].

The colorimeter was used to perform color tests, according to (Monalisa et al., 2022) on the color scale; the color meter displayed three (L* a * b) parameters that reflect the color value as indicated below [31]:

“L*” value defines the lightness of products if less than 50 indicates darkness and a value higher than 50 describes lightness.

**“a”** value defines the green or red color. The negative **“a”** value shows green while the positive indicates red color.

**“b”** value defines the blue or yellow color. The negative **“b”** value exhibits blue while positive indicates yellow color.

### Statistical analysis

In this study, different experiments were performed. The regression analysis was done to find the process curves for best-fit curve exponential decay. The analyses were implemented to compare different parameters at 5% level of confidence or significance.

## 3 Results and discussions

### 3.1 Zero load test

**Fig. 5:**
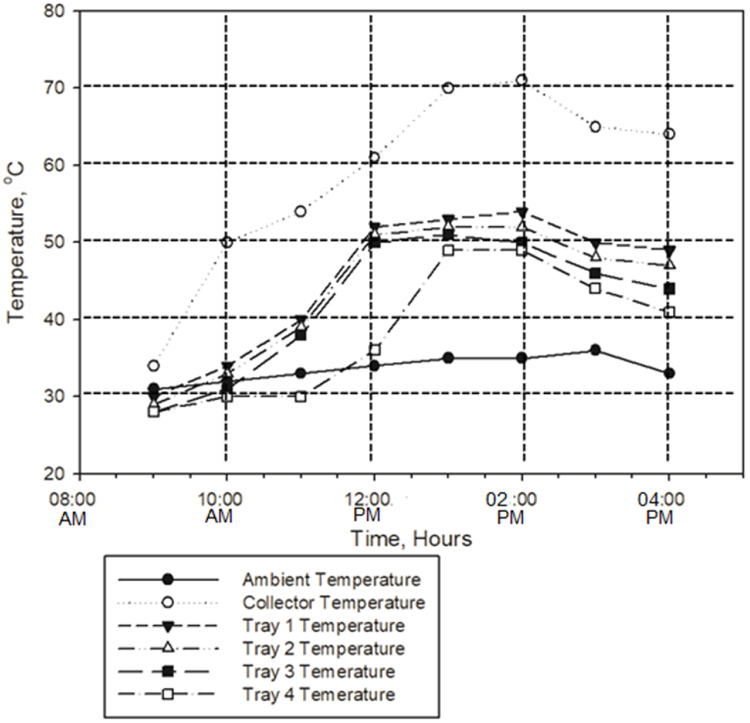
Results of zero load test results of the dryer.

Before starting the experiment, dryer performance was evaluated in terms of temperature variations. The temperatures of all four trays were recorded between the 8:00 a.m. and 4:00 p.m. while all three fans were running at full speed. Maximum temperature (71 °C) was recorded for the solar collector at 02:00 p.m. with an average collector temperature of 56 °C. The collector reached its peak temperature when the ambient temperature was 37 °C. The maximum temperature was recorded for tray 1, and the average tray temperature was 53 °C. Fig. 5 presents the change in temperature for solar collector and trays.

### 3.2 Drying apple slices with single fan operation

**Fig. 6:**
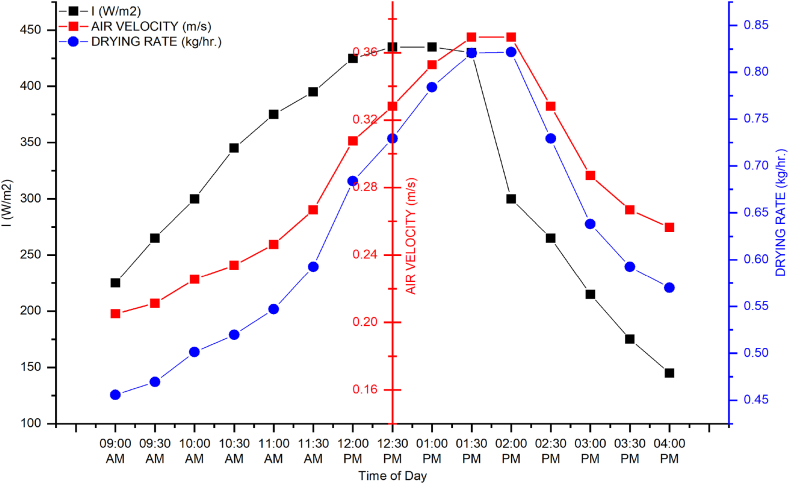
Dryer performance with one working fan.

The experiment was conducted in outdoor conditions. In the initial trial, 4 kg of apple slices were placed in the dryer, and only one fan in the middle of the dryer was turned on to ensure even flow. The results (Fig. 8) indicated that at the start of the experiment, the air velocity, solar irradiance, and drying rate were low from 8:00 a.m. to 11:00 a.m. After that, both sun irradiation and air velocity increased gradually. Because a photovoltaic module powered the fan, more power was available to the fan when solar irradiance increased, resulting in increased airflow. Results suggested that drying rate was associated with solar irradiance and air velocity. Energy contents were added through the transparent glass to trays, so the maximum drying rate was observed from 12:30 p.m. to 02:00 p.m. With the decrease in solar irradiance, air velocity and drying rate were also reduced. The maximum solar irradiance, air velocity, and drying rate values were 435 W/m^2^, 0.38 m/s, and 0.86 kg/h at 1:30 p.m., respectively. The minimum value of solar irradiance was 145 W/m^2^ at 4:00 p.m., and air velocity along with the drying rate was 0.2 m/s and 0.45 kg/h at 9:00 a.m. In the evening, the drying rate was higher than morning due to the whole day heating of the collector, as shown in Fig. 6.

### 3.3 Drying apple slice with two fan operations

**Fig. 7:**
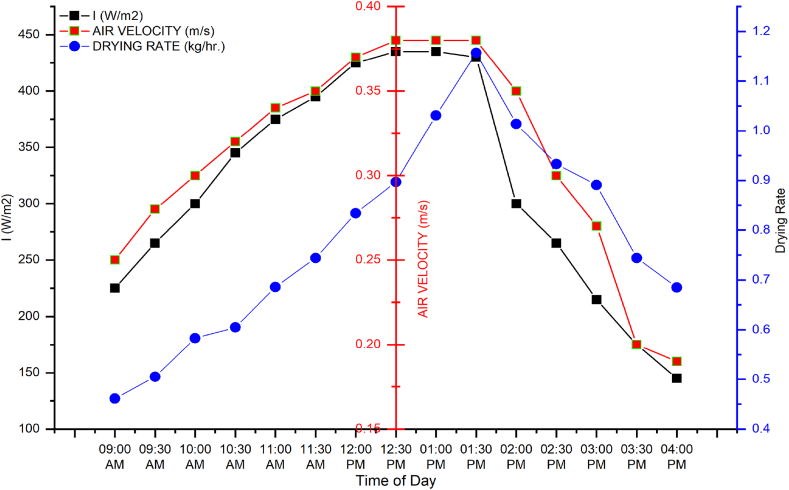
Dryer performance with two working fan.

Fig. 7 shows the drying rate and air velocity trend during the second trial when two fans, excluding the central fan, were switched on. The graph shows that the drying rate was lowest at 9:00 a.m. and increased gradually until it reached its maximum value at 1:30 p.m. A similar pattern was observed for solar irradiance and air velocity. The values were increased from 9:00 a.m. till they reached a maximum at 1:30 p.m. The maximum value for solar irradiance, drying rate, and air velocity was 430 W/m^2^, 1.157 kg/h, and 0.38 m/s, respectively, at 1:30 p.m. The minimum value for solar irradiance and air velocity was 147 W/m^2^ and 0.19 m/s recorded at 4:00 p.m., while for drying rate, the minimum value was 0.46 kg/h at 9:00 a.m. The air velocity was reduced in the evening due to the low availability of solar irradiance, while the drying rate was not at its lowest value compared to the morning because of the heat available in the heating section (see Table 2).

**Fig. 8:**
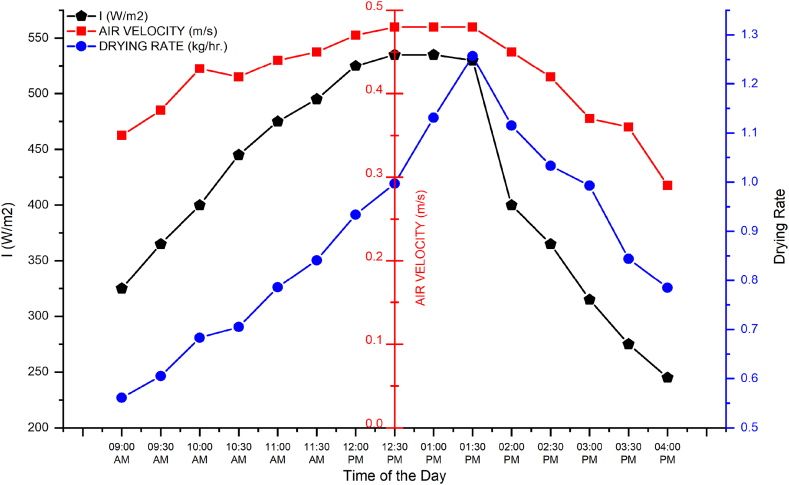
Dryer performance with three working fan.

#### 3.3.1 Drying apple slices with three fan operation

**Fig. 9:**
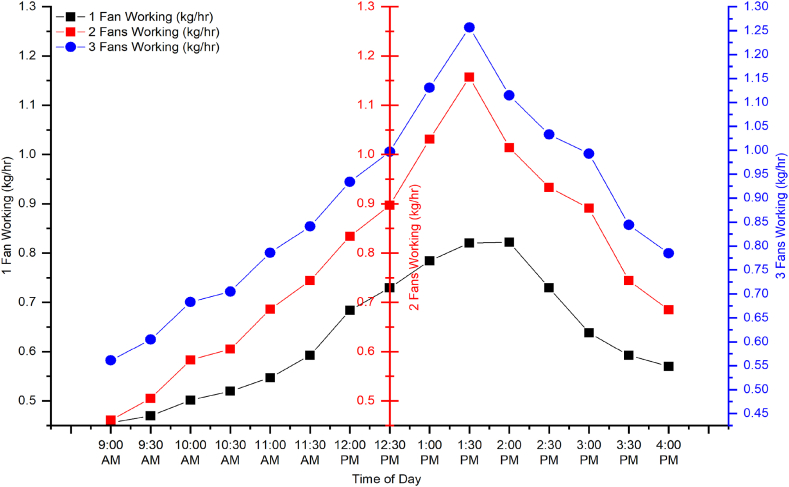
Comparison of drying rates for different fan working conditions.

Fig. 8 shows the drying rate and air velocity results when all three fans were switched on. The graph indicates that air velocity was higher than treatments when single or two fans were switched on. The simple reason was the working of all three fans. The results suggested that solar irradiance and drying rate increased from 9:00 a.m. and reached maximum at 1:30 p.m. The maximum value for solar irradiance, air velocity in the dryer, and drying rate was 535 W/m^2^, 0.48 m/s, and 1.251 kg/h at 1:30 p.m., respectively. The minimum value for solar irradiance and air velocity in the dryer was 245 W/m^2^ 0.29 m/s at 4:00 p.m., and the drying rate was 0.56 kg/h at 9:00 a.m. The drying rate at 4:00 p.m. was 0.785 kg/h, exceeding the minimum value of 0.56 kg/h in the morning. This was due to the same phenomenon that the collector area was heated throughout the day therefore although solar irradiance was less in the evening than morning time. Fig. 9 and Table 3 shows summery of experimental results.

**Table 3:** Summary of experiment for apple slices.

Sr. No.	Particular	1 fan working	2 fan working	3 fan working
1	Wet sample (g)	78.2	79.2	79.9
2	Dry matter (g)	8.7	8.6	8.6
3	Initial sample weight (g)	400	400	400
4	Final sample weight (g)	56	55	55
5	Initial moisture (%)	88.8	89.1	89.1
6	Final moisture (%)	16.6	16.2	15.8
7	Time to dry sample (h)	17.2	12.2	14.8

## 4 Results

**Fig. 10:**
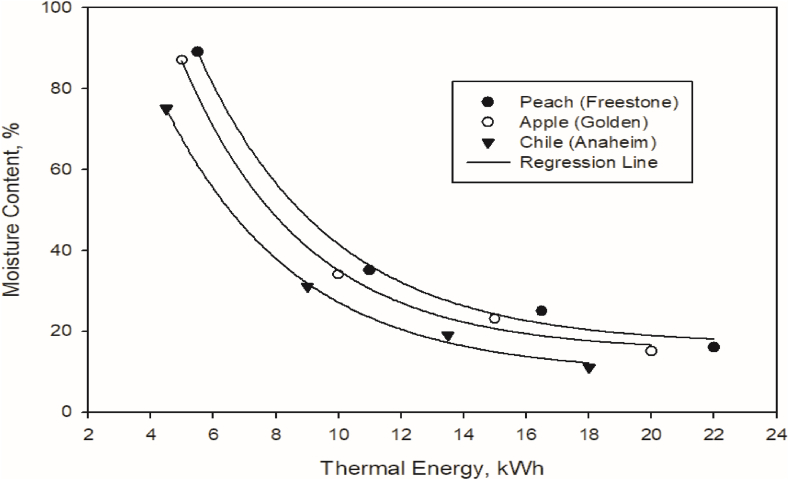
Effect of thermal energy on change in moisture content using the hybrid mixed mode solar dryer.

A hybrid mixed-mode solar dryer was used to dry the peach (Freestone), apple (Golden), and chili (Anaheim). The 10 kg of fresh perishable agricultural products were used for drying purposes after blanching. Nine different experiments were carried out. The initial moisture content was 89%, 88%, 90% for peach (Freestone), 87%, 88%, 86% for apple (Golden), and 74%, 76%, and 75% for chili (Anaheim), with an average moisture content of 89%, 87%, and 75% for peach (Freestone), apple (Golden), and chili (Anaheim), respectively. The average final moisture content use of average thermal energy calculations is shown in Table 4. The drying time of the hybrid mixed-mode solar dryer was compared with the other drying systems [13,27,32]. After comparison, it was found that the hybrid mixed-mode solar dryers need less drying time for similar agricultural products. The process curves, in terms of moisture content, as a function of thermal energy (kWh) are shown in Fig. 10.

**Table 4:** Drying yield during different experiments.

Sr. No.	Fruit	Sample weight	Drying time	Other systems drying time	M.C_I_	M.C_F_	Used thermal energy
		kg	Hrs	Hrs	%	%	kWh
1	Peach	10	17	20	89	16	22.2
2	Apple	10	16	19	87	15	20.3
3	Chili	10 14		28	74 11		18.5

Regression analysis was used to fit the best curve for the percentage of moisture content decrease data as a function of thermal energy required during the complete drying process. The best fit curve for all perishable agricultural products was exponential decay, as shown in Eq. (12).

(12) $$F\left(d\right)=C+xe^{-yd}$$

where % moisture content decreasing *F(d)* is a function of variable thermal energy *d* and *C* is the offset of moisture removal curve, *x* and *y* are the fitted parameters, *e* is exponential constant with a value of 2.71 in the regression analysis. The accuracy of the regression analysis was assessed by experimental data and drying curves, shown in Fig. 10. The regression analysis parameters and coefficient of determination are given in Table 5.

**Table 5:** Regression analysis parameters and coefficient of determination (R^2^).

Sr. No.	Fruits	Parameters of regression analysis			R^2^
		*C*	*x*	*y*	
1	Peach	16.5	265.4	0.23	0.99
2	Apple	15	258.5	0.25	0.98
3	Chili	09	190.8	0.23	0.99

The experimental findings revealed high R^2^ values, indicating a regression analysis with the greatest fit. The experimental results showed that using the same amount of thermal energy throughout the drying process, a higher moisture removal rate in the early stages and a very low moisture removal rate at the end of the drying process.

### 4.1 Quality analysis of dried products

Proximate analyses identify each dried sample composition. The compound identification for peach, apple, and chili is shown in Table 6.

**Table 6:** Chemical constituents of dried peach, apple, and chili.

Sr. No	Fruit	M.C	Ash	Crude Protein	Fat	Fiber	Carbohydrates
		%	%	%	%	%	%
1	Peach	16.5	3	2.5	18	0	60
2	Apple	15.4	2.2	0.04	2.87	0	79.4
3	Chili	11	1.1	2	1.5	8.5	75.9

The low moisture content indicates no fungal attack on the dried sample since fungi require more than 80% moisture content for normal growth. In addition, each sample has shown a low percentage of ash, crude protein, fat and fibre and a high percentage of carbohydrates, indicating that the quality of dried perishable agricultural products lies within the standard food range and is appropriate for powder processing and can be put on sale.

TPC test for peach, apple, and chili were 1.9 × 10^4^ cfu/g, 1.2 × 10^4^ cfu/g, and 1.7 × 104 cfu/g bacteria, respectively (cfu/g = colony-forming unit per gram of sample) bacteria was within the safe limits in the dried sample.

The color test of dried peach, apple, and chili is shown in Table 7.

**Table 7:** Color analysis of dried peach, apple, and chili using the colorimeter.

Sr. No.	Fruit	L*	a	b	Sample color
1	Peach	53	11	32	Light golden
2	Apple	59	14	44	Light red and yellow
3	Chili	28	25	17	Deep red

The quality analysis results indicate that solar drying does not affect the sample chemical constituents, total bacteria and color, and the dried samples quality was within the recommended values of quality food standards.

### 4.2 Economics analysis

An annual economic study of a hybrid mixed-mode solar dryer was conducted annually. The total investment was $500, including fabrication and materials (heating, mixed mode drying, PV panel, battery, and so on). In terms of beginning cost and income, the break-even threshold was determined as follows:

T_I_ = T_Cost_

Y × X = T_FC_ + V × X

$$X=\frac{T_{FC}}{Y-V}$$

$$X=\frac{64}{4.3-3.9}$$

X = 160 days

where, T_I_ is the total income, T_Cost_ is the total cost, Y is the income/hour based on the price of dried product, V is the variable cost (labor and maintenance cost), T_FC_ is the total fixed cost (interest on financing and labor benefits). X is the total operating time in hours.

The 10 kg (total price of $10) of apple (Golden) was used for drying, 1.5 kg of dried apple powder was taken in 16 h. If 1 kg dried apple powder was sold at $16 (99.99% Pure), then $14 can be produced daily.

Note: Income $14 = total sells ($16+ $8) – total initial cost $ 10.

**Fig. 11:**
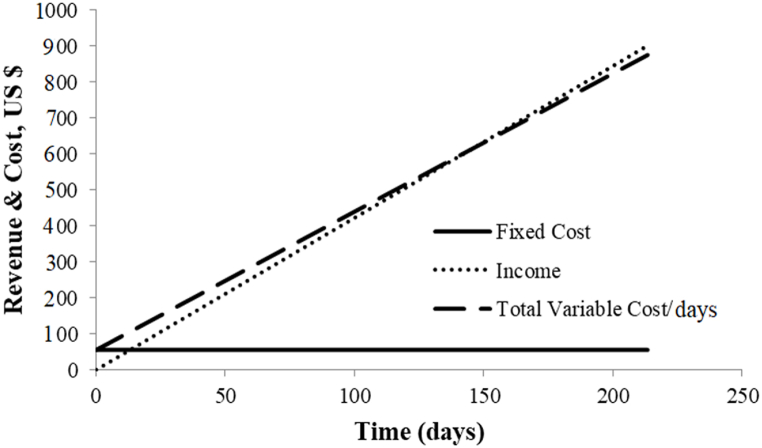
Break even analysis (1 day = 16 h).

The result of the break-even point for the hybrid mixed-mode solar dryer is 160 days, as shown in Fig. 11. Furthermore total number of sunny days in Pakistan and neighboring countries ranges from 185 to 290 per year [33]. According to the break-even analysis, the developed hybrid mixed-mode solar dryer has no risk factor. Furthermore, because the system functions at a low cost, only revenue will be generated at the end of the payback period.

## 5 Conclusions

• The hybrid mixed-mode solar dryer was designed, developed and tested at PMAS-Arid Agriculture University Rawalpindi.
• The average heating section temperature, mixed mode drying section temperature, ambient temperature, and sun intensity were 65 °C, 57 °C, 32 °C, and 780 W/m^2^, respectively, without load condition.
• The peach (Freestone), apple (Golden), and chili (Anaheim) need 17-14 h for complete drying.
• The average efficiency of the system was 33%.
• According to the quality analysis, the sample composition, total bacteria, and color were all within the recommended quality food criteria.
• It was concluded that the system's total cost and payback duration were $500 and 0.44 years, respectively. The costs and payback periods for the other systems [[19], [20], [21]] were $670, $2361, $4600, and 0.65, 1.5–2.1, and 1.89 years, respectively.
• As a result, the solar mixed-mode solar dryer appears to be a viable option for drying various perishable agricultural items. Solar-based renewable technology has a negligible operating cost and can help advance the field of post-harvest technology significantly.

## Author contribution statement

Arslan Afzal: Wrote the paper; Analyzed and interpreted the data; contributed reagents, materials, analysis tools or data.

Tahir Iqbal, Kamran Ikram: Analyzed and interpreted the data; Conceived and designed the experiments.

Muhammad Naveed Anjum: Wrote the paper; Contributed reagents, materials, analysis tools or data.

Muhammad Azam, Muhammad Umair, Fiaz Hussain: Analyzed and interpreted the data; Conceived and designed the experiments.

Sajeela Akram: Conceived and designed the experiments; Wrote the paper.

Muhammad Ameen ul Zaman, Abid Ali: Performed the experiments; Conceived and designed the experiments.

Faizan Majeed: Wrote the paper, Analyzed and interpreted the data.

## Funding statement

This work was supported by the Data Driven Smart Decision Platform [DDSDP-332].

This work was supported by 10.13039/501100010221Higher Education Commission of Pakistan [2347].

## Data availability statement

Data will be made available on request.

## Declaration of interest’s statement

The authors declare no conflict of interest.
